# Identification and Potential Functions of MicroRNAs Involved in Freshwater Adaptation in Aquatic Firefly *Aquatica leii*

**DOI:** 10.3390/insects17080845

**Published:** 2026-08-14

**Authors:** Shi-Ling Wang, Yan-Hong Chen, Li-Ying Yang, Lian-Bing Lin, Wei-Wei Li, Qi-Lin Zhang

**Affiliations:** 1 Faculty of Life Science and Technology, Kunming University of Science and Technology, Kunming 650500, China; 2 Yunnan Key Laboratory of Biodiversity Information, Kunming Institute of Zoology, The Chinese Academy of Sciences, Kunming 650201, China; 3 Yunnan International Joint Center of Urban Biodiversity, Kunming 650201, China

**Keywords:** aquatic firefly, miRNAomics profiles, fresh water, regulatory mechanisms

## Abstract

Aquatic insects are well-adapted to freshwater habitats; however, the contribution of miRNA-mediated post-transcriptional regulation to freshwater adaptation remains unexplored. Here, 12 miRNAomes for larval and adult aquatic (*Aquatica leii*) and terrestrial (*Pyrocoelia praetexta*) fireflies were generated for comparative analyses. In total, 20 differentially expressed miRNAs (DEMs) specific to *A. leii* were screened as aquatic adaptation-related miRNAs via intraspecific and interspecific comparisons. A functional enrichment analysis of target genes showed that these miRNAs significantly (false discovery rate, *FDR* < 0.05) regulate 10 pathways associated with key biological processes, such as energy metabolism (oxidative phosphorylation, 2-oxocarboxylic acid metabolism, pantothenate and CoA biosynthesis, valine, leucine and isoleucine biosynthesis, and insulin secretion pathway), defense/detoxification response (drug metabolism—cytochrome P450), neural signal transduction (serotonergic synapse and sphingolipid metabolism), as well as development and lifespan (hedgehog signaling pathway-fly and longevity regulating pathway). A miRNA–target–pathway network uncovered a broad post-transcriptional regulatory mechanism related to freshwater adaptation in fireflies. The roles of two randomly selected miRNA–mRNA pairs were validated through in vivo and in vitro experiments using miRNA mimics. The current study is the first to explore a miRNA-mediated potentially regulatory network for aquatic adaptation in fireflies and even insects, providing key insights into miRNA-mediated post-transcriptional regulation contributing to survival in freshwater environments.

## Simple Summary

So far, little is known about the regulatory roles of microRNA (miRNA) in aquatic adaptation in insects. How aquatic insects adapt to freshwater environments remains largely unknown. According to larval living habits, fireflies (Coleoptera: Lampyridae) include terrestrial, semi-aquatic, and aquatic lineages, making them an excellent model for investigating the aquatic adaptation of insects. This study generated miRNAomics profiles of two firefly species (aquatic *Aquatica leii* and terrestrial *Pyrocoelia praetexta*, covering the respective adults and larvae). A set of aquatic adaptation-related miRNAs (AARmiRNAs) and the significantly regulated pathways were further revealed via intraspecific and interspecific comparative miRNAomics as well as functional enrichment analysis of potential target genes of AARmiRNAs. These AARmiRNAs were primarily associated with energy metabolism, defense/detoxification response, neural signal transduction, as well as development and lifespan. This study expands the understanding of the regulatory roles of miRNAs in aquatic adaptation in insects.

## 1. Introduction

Aquatic insects, generally defined as those that inhabit freshwater habitats during one or more life stages [1], have successfully adapted to physiological challenges related to respiration, predation, locomotion, and other processes in fresh water [2]. Several studies have evaluated the molecular mechanisms underlying insect adaptation to aquatic environments, such as a lineage-specific amino acid substitution at several mitochondrial COI sites in freshwater insects, relative to terrestrial species [3,4]. However, the molecular regulatory mechanisms underlying the adaptation of insects to fresh water remain largely unexplored, particularly the roles of non-coding microRNAs (miRNAs). miRNAs, as endogenous non-coding RNA molecules that suppress gene expression, generally bind to the 3′ untranslated region of the target genes, playing key roles in responding to environmental stress [5,6,7]. Several studies have focused on miRNAs to elucidate the mechanisms underlying the adaptation of animals (particularly fish, shellfish, and crustaceans) to aquatic environments [5,6,7,8]. For example, six miRNAs (e.g., miR-2353, miR-1984, and miR-183) were found to be associated with osmotic stress of seawater in marine bivalves (*Crassostrea* species) [7]. MiRNA molecules, miR-998-y and miR-275-z, identified from the mRNA–miRNA regulatory network were considered as potential biomarkers for hypoxia response in crabs (*Eriocheir sinensis*) [8]. These studies have demonstrated that miRNAs play a pivotal role in the ecological adaptation of animals to aquatic environments; however, little is known about their roles in insects.

Based on their habitat selection, firefly (Coleoptera: Lampyridae) larva can be categorized into three distinct types: terrestrial, semi-aquatic, and aquatic lineages [9]. Compared to the terrestrial relatives, aquatic fireflies face great physiological challenges in freshwater environments. For example, only approximately 1/20 of air oxygen content is available to aquatic lineages [10]. Compared to terrestrial and semi-aquatic lineages, aquatic fireflies have developed numerous biological structures and life strategies (e.g., up to eight types of swimming behavior, branching tracheal gills, and smoother body surface textures) to adapt to these ecological stresses in fresh water (e.g., hypoxia, water pressure and resistance, and decreased lighting) [11,12,13,14]. Therefore, fireflies are useful experiment models for investigations of the molecular adaptations of insects to freshwater habitats. Furthermore, molecular mechanisms have been investigated through comparative transcriptomic and metabolic analyses, uncovering several significant adaptive signals associated with metabolic efficiency and aquatic-specific morphological features (e.g., cuticle chitin, tracheal system, and compound eye) [15,16]. Although the potential mechanisms underlying the adaptation of fireflies to fresh water have been explored [15,16], the miRNAs that orchestrate freshwater adaptation in fireflies and their mechanisms of action are unknown.

**Figure 1:**
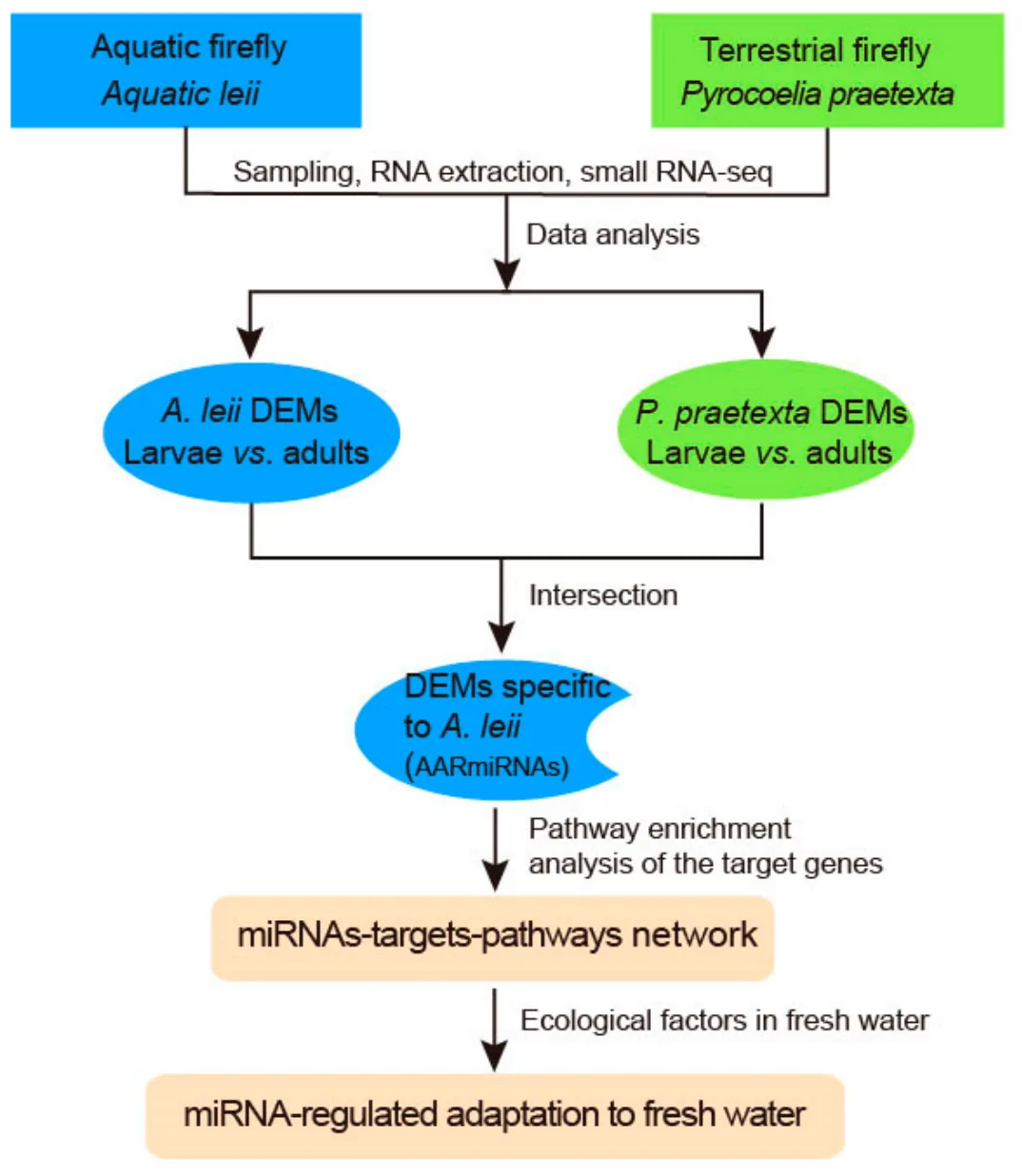
Flow chart of experiments and data processes. DEMs indicate differentially expressed miRNAs. AARmiRNAs indicate aquatic adaptation-related miRNAs. Dark blue, green, and light orange indicate processes implemented in aquatic firefly *A. leii*, terrestrial firefly *P. praetexta*, and AARmiRNAs, respectively.

The present study employed larvae and adults of two typical aquatic and terrestrial firefly species, including *Aquatica leii* and *Pyrocoelia praetexta* (belonging to subfamily Luciolinae, with completely aquatic and terrestrial larval stages, respectively) [15]. They show complete metamorphosis including egg, larval, pupal, and adult stages. The developmental period of larvae of both species is the longest and lasted approximately 300 days. The lifecycle of these two species and their distinctions were illustrated in Zhang et al. [15]. Next, miRNAomes of larvae and adults of those two firefly species were generated using high-throughput small RNA sequencing (sRNA-seq). We conducted intraspecific and interspecific comparisons of the adult and larval miRNA profiles of both species. Next, miRNAs with significant changes specific to *A. leii* (namely, aquatic adaptation-related miRNAs (AARmiRNAs)) were identified by comparing differentially expressed miRNAs (DEMs) between both species. Subsequently, the *A. leii* transcriptome assembled in a previous study [15] was employed to provide reference data for the identification and pathway enrichment analyses of target genes of AARmiRNAs (Figure 1). This study uncovered regulatory networks (miRNAs–targets–pathways) contributing to firefly adaptation to freshwater environments.

## 2. Materials and Methods

### 2.1. Sampling

*Pyrocoelia praetexta* and *A. leii* individuals were maintained in the laboratory of the Faculty of Life Science and Technology, Kunming University of Science and Technology, Kunming, China, as described in Zhang et al. [15]. Sample collection was conducted according to previously described methods [15,16]. In particular, *A. leii* and *P. praetexta* individuals were randomly collected to obtain 10 individuals for each of 4th and 5th instar larvae and female and male adults. The sampling was repeated independently three times to yield three biological replicates. The samples were put into a 10 mL RNase-free centrifugal tube and stored at −80 °C until use.

### 2.2. RNA Extraction, Library Construction, and Sequencing

The whole-body samples from larval and adult stages were separately homogenized in 2 mL of TRIzol (Invitrogen, Carlsbad, CA, USA) per 50–100 mg of tissue. Details of RNA extraction were presented in Supplementary File. To avoid gene expression variation as a result of different larval instars and adult stages as well as to adequately cover genes at each larval and adult stage [15], the total RNA samples from each of the two larval stages were pooled in equal amounts to generate the total RNA for the larval stage. RNA samples from female and male adults were similarly pooled. The total mixed RNA was obtained separately for larvae and adults of each firefly species.

The libraries used for sRNA-seq were constructed using the Small RNA-Seq Library Prep Kit (Lexogen, Vienna, Austria) (Supplementary File). In total, 12 small RNA-sequencing libraries were generated, referred to as ALA1–3 (*A. leii* adults 1–3), ALL1–3 (*A. leii* larvae 1–3), PPA1–3 (*P. praetexta* adults 1–3), and PPL1–3 (*P. praetexta* larvae 1–3). These libraries were sequenced using the HiSeq 2500 platform (Illumina, San Diego, CA, USA) to generate single-end 50 bp reads at Beijing Genomics Institute (BGI, Shenzhen, China) under BGI quality control.

### 2.3. Data Preprocessing

The raw tags generated by Illumina sequencing were filtered to remove poly-N, adaptors, and low-quality reads, resulting in the retention of clean tags with lengths ranging from 18 to 30 bp. Subsequently, non-coding sRNAs (e.g., rRNAs, tRNAs, and snoRNAs) were annotated through BLAST searches (version 2.0, Bethesda, MD, USA) against the GenBank and Rfam v15.0 databases [17]. The non-coding sRNAs that were annotated were excluded from further analyses. RepeatMasker v4.2.1 (http://www.repeatmasker.org/, access date: 27 August 2025) was employed to screen and eliminate highly repetitive sequences and low complexity sequences within the clean tags.

### 2.4. Identification of Known MiRNAs and Differential Expression Analysis

In order to identify known miRNAs, the above obtained clean tags were matched to known miRNA precursors and mature miRNAs in the miRBase v22.1 database (https://ngdc.cncb.ac.cn/databasecommons/database/id/165, access date: 1 August 2025) [18], without mismatches employed according to previous studies [19,20]. The miRNAs shared by the three biological replicates belonging to each group were reserved for further analysis.

MiRNA expression levels were quantified according to transcripts per million (TPM) values. A principal component analysis (PCA) was performed using a TPM-based R procedure (*FactoMineR*v2.11, Rennes, France) to assess correlations among the biological replicates. Certain miRNAs (more than two mapped tags) were either not detected or only detected in larval or adult groups for each firefly species, suggesting that the expression of these miRNAs was affected by the stage. To include these in the identification of candidates for freshwater adaptation, the union of recognized miRNAs in the larval and adult samples was utilized to screen DEMs. The current analysis used a more stringent filtering criterion than those in previous studies [21,22] for the identification of DEMs to avoid false-positive results. The thresholds for DEM identification were both |Log2 fold change (FC)| >1 and adjusted *p*-value (false discovery rate, *FDR*) <0.05. The significance of each DEM between larvae and adults of each firefly species was evaluated using *DESeq2* (v1.34.0) [23]. Moreover, low-abundance miRNAs are prone to large differences among sequencing experiments; miRNAs with TPM values less than 0.5 in the larval or adult groups were thus excluded from subsequent analyses [24].

### 2.5. Identification of Aquatic Adaptation-Related miRNAs (AARmiRNAs) in Aquatic A. leii Larvae

Identification methods for AARmiRNAs in larval *A. leii* have been described in earlier studies [15,16]. In brief, the DEMs identified between adult and larval stages of *P. praetexta* were intersected with DEMs of *A. leii* to remove miRNAs for *A. leii* larvae that were not involved in freshwater adaptation. Subsequently, DEMs found only in *A. leii* (*A. leii* species-specific DEMs) were retained as miRNAs whose abundance changes in response to freshwater environments. These miRNAs were thus identified as AARmiRNAs in *A. leii* larvae.

### 2.6. Kyoto Encyclopedia of Genes and Genomes (KEGG) Enrichment Analysis of Target Genes of AARmiRNAs, Construction of Regulatory Networks, In Vivo and In Vitro Determination of miRNA–mRNA Relationship

To gain a deeper understanding of the biological functions of AARmiRNAs associated with aquatic adaptation, the functions of target genes were predicted using *RNAhybrid* [25] v2.1.2 and *miRanda* [26] v3.3 software. The target genes shared by these two tools were retained for analyses. The target gene sequences were aligned using BLAST [27] and annotated against NCBI RefSeq, KEGG, and UniProt databases. The hypergeometric test was applied to determine KEGG pathways significantly enriched for target genes corresponding to miRNAs compared with the entire genomic background. *FDR* values of <0.05 were considered statistically significant. A miRNA–target–KEGG pathway network was constructed based on the targeting relationships of miRNAs to mRNAs and KEGG pathways associated with the corresponding targets. The target genes annotated to “hypothetical protein” or unknown function and with no annotation were excluded.

Three miRNA–mRNA pairs (miR-279e-3p-*galt*, miR-1b-3p-*gpt*, and miR-1b-3p-*tret1*) were chosen randomly to verify miRNA regulation on the corresponding target genes through in vivo experiments using agomir and antagomir mimics of miRNAs in *A. leii*, as employed in our recent studies [28]. Agomir, antagomir, agomir negative control (Ago_N.C.), and antagomir negative control (Ant_N.C.) were synthesized by Sangon Biotechnological Co. (Shanghai, China). Ten 4th instar *A. leii* larvae were randomly selected, and miRNA agomir mimics (1 μL, 40 μM) were administered in each individual by injection into the ventral abdomen using a microsyringe (Shanghai Bolige Industry & Trade Co., Ltd., Shanghai, China). Similarly, antagomir mimics and N.C. mimics of miRNAs were separately injected into larvae *A. leii* and set controls without any treatment. The experiments were repeated three times as different biological replicates. After 48 h of injection, a similarly employed treatment time in other beetles [29], the experimental samples were collected to test the expression levels of target genes and miRNAs using quantitative real-time PCR (qRTPCR) (Supplementary File and Supplementary Table S1).

A dual-luciferase assay was employed to further verify the three miRNA–mRNA pairs through in vitro experiments. Briefly, the specific primers with the XhoI and SacI restriction sites of *galt*, *gpt*, and *tret1* were designed based on the predicted miRNA–mRNA binding site and their flanking sequences (Supplementary Table S2). Subsequently, PCR amplification was performed to obtain PCR products. Wild-type (WT) fragments containing the predicted miRNA binding site were inserted into the pmirGLO vector (Promega, Madison, WI, USA) via the restriction sites and T4 DNA ligase; finally pmirGLO-*galt*-WT, pmirGLO-*gpt*-WT, and pmirGLO-*tret1*-WT were obtained. Meanwhile, mutant (MUT) fragments containing the predicted miRNA binding site were synthesized via Beijing Tsingke Biotech Co., Ltd. (https://www.tsingke.com.cn/, Beijing, China) and inserted into the pmirGLO vector, resulting in pmirGLO-*galt*-MUT, pmirGLO-*gpt*-MUT, and pmirGLO-*tret1*-MUT. The empty pmirGLO vector served as a negative control. Next, HEK293T cells were co-transfected with 0.25 µL of either the agomir or the Ago_N.C. (including 100 ng of WT-pmirGLO, MUT-pmirGLO, or empty pmirGLO plasmid and 1 μL of Lipofectamine 3000 (Invitrogen, CA, USA) transfection reagent) in a 25 µL reaction system. At 24 h post-transfection, 75 μL of Dual-Glo^®^ Luciferase Reagent was added to the cells and then Stop & Glo^®^ Reagent was added. Finally, the activities of firefly luciferase and *Renilla* luciferase were determined using a Varioskan LUX microplate reader (Thermo Fisher, Waltham, MA, USA).

## 3. Results

### 3.1. Overview of Sequencing Data

Twelve small RNA libraries were sequenced for larvae and adults of *A. leii* and *P. praetexta* using sRNA-seq technology. We obtained raw tags ranging from 9.8 to 12.4 million per library. Low-quality sequences were filtered out, and clean tags accounting for 45.36% to 95.24% of the raw data were obtained from each library (Supplementary Table S3). The lengths of clean tags in all libraries were predominantly concentrated within the range of 18–24 nt, as similarly reported for other insects [30,31].

The PCA revealed distinct library clusters corresponding to each firefly species and developmental stage, with variation among groups (Supplementary Figure S1). This observation revealed a high degree of similarity among three biological replicates and discernible differences among the four groups, demonstrating robust sampling and sRNA-seq processes.

### 3.2. Identification of Known and Differentially Expressed MiRNAs

For the three biological replicate libraries generated from PPL, 184, 183, and 180 known miRNAs were identified, with 150 miRNAs shared by all of the libraries. Additionally, 183, 201, and 191 known miRNAs were identified for PPA, including 155 that were shared by all three libraries. In ALL, 186, 182, and 180 known miRNAs were identified in three libraries, 149 of which were shared by all of the libraries. For ALA, 186, 191, and 203 known miRNAs were identified in three sequencing libraries, and 162 miRNAs were shared by all three libraries.

**Figure 2:**
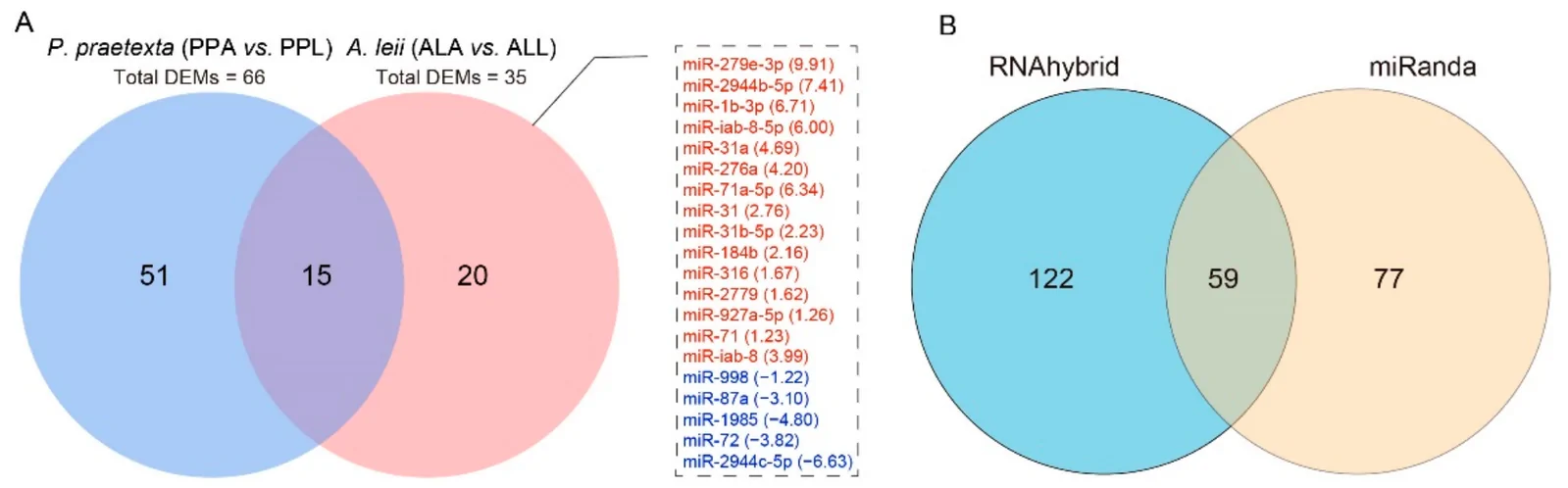
Venn diagram of differentially expressed miRNAs obtained from *P. praetexta* (adults vs. larvae, a light purple solid circle) and *A. leii* (adults vs. larvae, a light pink solid circle), and in the dashed rectangular box on the right, the up- and down-regulated miRNAs were highlighted with red and blue colors, respectively, with fold change (ALA/ALL) (**A**). Venn diagram of number of AARmiRNA target genes predicted by RNAhybrid (light blue) and miRanda (light orange) (**B**).

Among these miRNAs, 35 DEMs were found in ALA vs. ALL, and 66 DEMs were observed in PPA vs. PPL (Figure 2A). These DEMs identified from aquatic and terrestrial lineages, respectively, were subsequently employed for AARmiRNA screening.

### 3.3. Identification of Aquatic Adaptation-Related MiRNAs

Among the DEMs identified in aquatic and terrestrial lineages, a total of 20 DEMs are species-specific to *A. leii* (identified as AARmiRNAs), including 15 up-regulated and five down-regulated miRNAs (Figure 2A and Supplementary Table S4). Among these AARmiRNAs (Figure 2A), eight miRNAs were highly up-regulated (i.e., miR-279e-3p, miR-2944b-5p, miR-1b-3p, miR-71a-5p, miR-iab-8-5p, miR-31a, miR-276, and miR-iab-8) in response to freshwater environments, with a change of more than 8-fold (|log2 fold-change| > 3), while four miRNAs were acutely down-regulated (i.e., miR-87a, miR-72, miR-1985, and miR-2944c-5p), with a change of more than 8-fold (|log2 fold-change| < −3) (Supplementary Table S4).

### 3.4. Network Analysis of MiRNAs–Targets–KEGG Pathways Contributing to the Adaptation of Aquatic A. leii to Fresh Water

In total, 181 and 136 target genes of AARmiRNAs were identified using *RNAhybrid* and *miRanda*, respectively (Figure 2B). Among these genes, 59 targets identified using both algorithms were considered the final set and were included in further analyses. The final target genes of AARmiRNAs were significantly enriched in 10 KEGG pathways (Supplementary Table S5). Among these KEGG pathways, five were primarily involved in energy metabolism, including 2-oxocarboxylic acid metabolism (ko01210), oxidative phosphorylation (ko00190), pantothenate and CoA biosynthesis (ko00770), valine, leucine and isoleucine biosynthesis (ko00290), and insulin secretion (ko04911). One pathway was associated with defense (detoxification)-related pathways, i.e., drug metabolism—cytochrome P450 (ko00982). Two pathways were associated with neural signal transduction, namely serotonergic synapse (ko04726) and sphingolipid metabolism (ko00600). Two KEGG pathways were involved in development and lifespan, including hedgehog signaling pathway-fly (ko04341) and longevity regulating pathway (ko04211).

**Figure 3:**
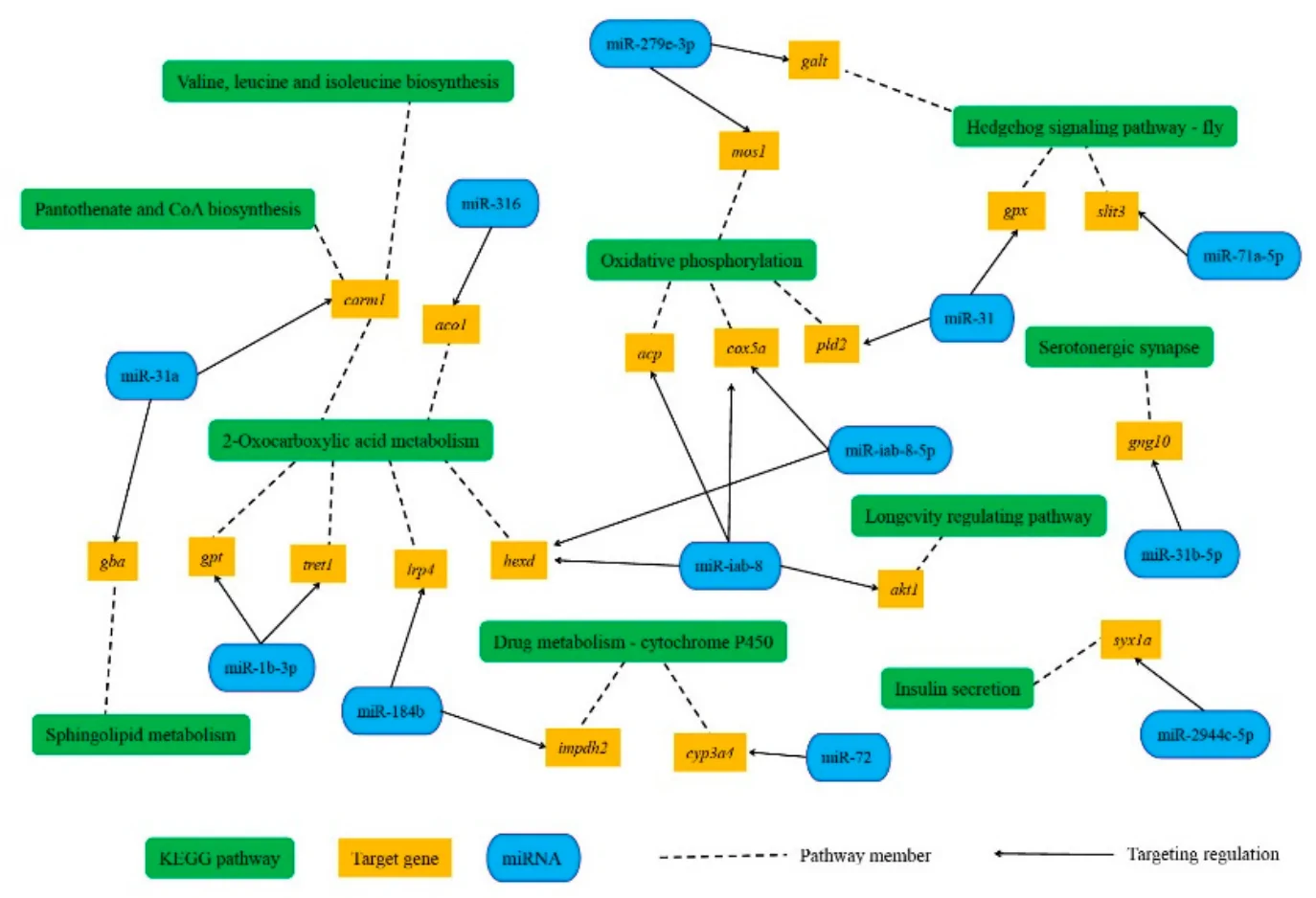
The AARmiRNAs–targets–KEGG pathway regulatory network. The AARmiRNAs are represented by the sky-blue boxes, the targeted genes by the light-orange boxes, and the KEGG pathways by the green boxes. The dotted lines connect the pathways to their associated target gene members; the arrows indicate that the target genes are regulated by AARmiRNAs. Note: *carm1*: coactivator-associated arginine methyltransferase 1 (c125669.graph_c0); *galt*: galactose-1-phosphate uridyltranserase (c132225.graph_c1); *slit3*: slit homolog 3 protein (c128654.graph_c1); *gpx1*: glutathione peroxidase (c75444.graph_c0); *akt1*: RAC serine/threonine-protein kinase (c129974.graph_c0); *syx1a*: syntaxin 1A (c125447.graph_c0); *gng10*: guanine nucleotide-binding protein, gamma 10 (c52838.graph_c0); *gba*: glucosylceramidase (c118608.graph_c1); *cyp3a4*: cytochrome P450 3A4 (c177762.graph_c0); *impdh2*: inosine monophosphate dehydrogenase 2 (c146647.graph_c0); *mos1*: mariner Mos1 transposase (c82057.graph_c0); *acp*: acyl carrier protein (c160089.graph_c0); *pld2*: phospholipase D2 (c156312.graph_c0); *cox5a*: cytochrome c oxidase subunit Va (c92461.graph_c0); *gpt*: alanine aminotransferase (c46235.graph_c0); *lrp4*: low-density lipoprotein receptor-related protein 4 (c121735.graph_c0); *tret1*: facilitated trehalose transporter Tret1 (c126480.graph_c0); *aco1*: probable cytoplasmic aconitate hydratase (c134062.graph_c0); *hexd*: hexosaminidase D (c130593.graph_c1).

**Figure 4:**
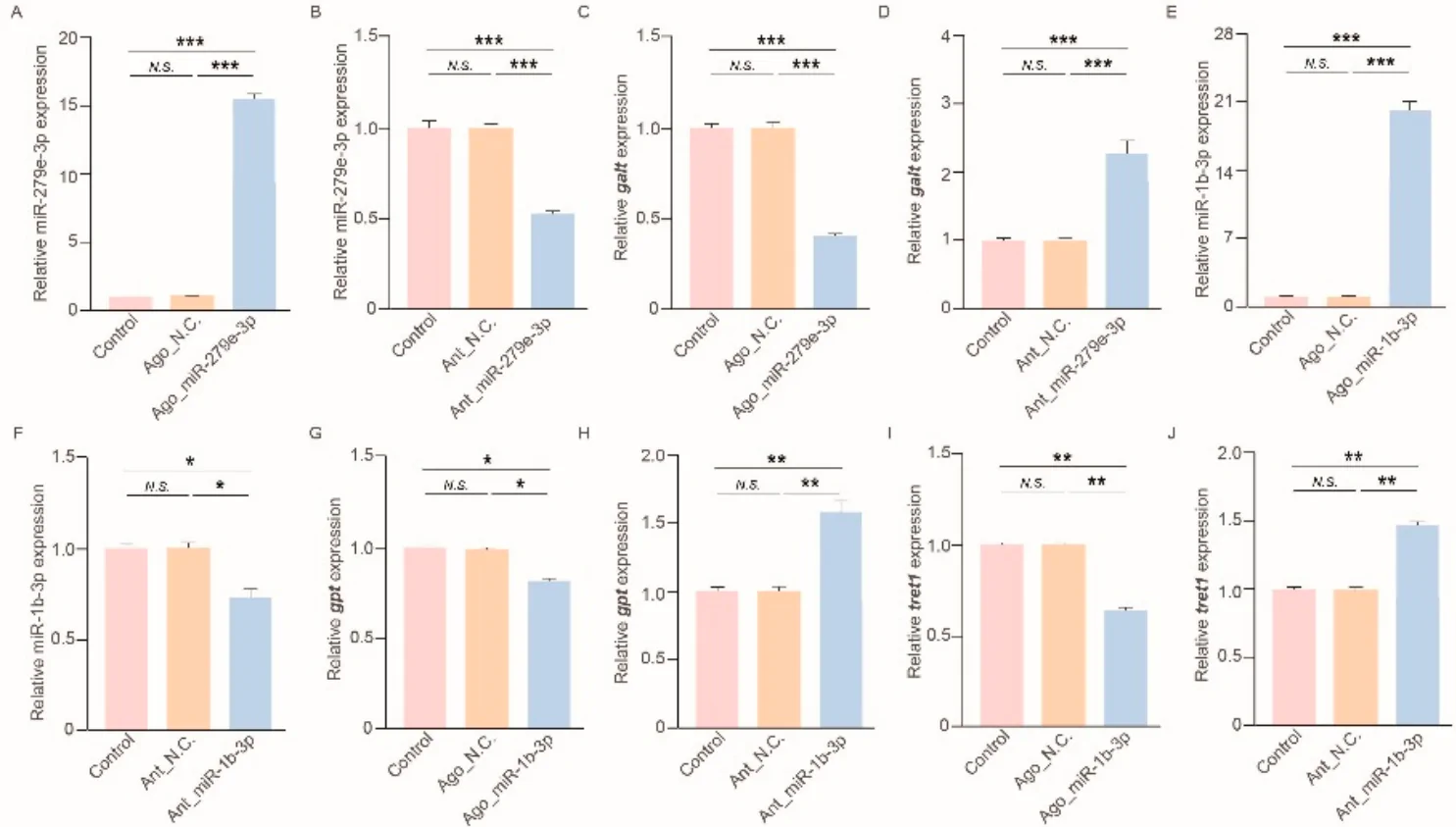
Effect of miR-279e-3p on *galt* expression (**A**–**D**) and effect of miR-1b-3p on *gpt* and *tret1* expression (**E**–**J**). (**A**) Expression levels of miR-279e-3p after treatment of Ago_miR-279e-3p. Expression levels of miR-279e-3p after treatment of Ant_miR-279e-3p (**B**). Expression levels of *galt* after treatment of Ago_miR-279e-3p and Ant_miR-279e-3p, respectively (**C**,**D**). Expression levels of miR-1b-3p after treatment of Ago_miR-1b-3p (**E**). Expression levels of miR-1b-3p after treatment of Ant_ miR-1b-3p (**F**). Expression levels of *gpt* after treatment of Ago_miR-1b-3p and Ant_miR-1b-3p, respectively (**G**,**H**). Expression levels of *tret1* after treatment of Ago_miR-1b-3p and Ant_miR-1b-3p, respectively (**I**,**J**). All data are presented as mean ±S.E. (*n* = 9). Significance values were calculated for each comparison pair using *t*-test. * represents *p* < 0.05, ** indicates *p* < 0.01, *** indicates *p* < 0.001 and *N.S.* denotes no significance.

The enriched KEGG pathways for the targets were used to construct a regulatory miRNA–mRNA–pathway network related to the adaptation of *A. leii* to freshwater habitats (Figure 3). After removing the target genes that could not be annotated or annotated to hypothetical protein, 12 AARmiRNAs and 19 corresponding targets were included in the regulatory network. Moreover, a table was created to highlight the miRNAs–target–pathways (Table 1), showing 2-oxocarboxylic acid metabolism regulated by the most of AARmiRNAs in the network, followed by oxidative phosphorylation. After injection of Ago_miR-279e-3p and Ago_miR-1b-3p in the network, expression levels of these two AARmiRNAs significantly (*p*-values < 0.05) increased compared to the controls and Ago_N.C., without significant difference between Ago_N.C. and the blank controls (Figure 4). Meanwhile, *galt* as target genes of miR-279e-3p and *gpt* and *tret1* as target genes of miR-1b-3p presented significant (*p*-values < 0.05) downregulation. Conversely, expression of the two AARmiRNAs was significantly (*p*-values < 0.05) lower in Ant_miR-279e-3p and Ant_miR-1b-3p groups compared to the controls and Ant_N.C. Expression of genes targeted by these two miRNAs significantly (*p*-values < 0.05) up-regulated relative to that of Ant_N.C. and the blank controls.

**Figure 5:**
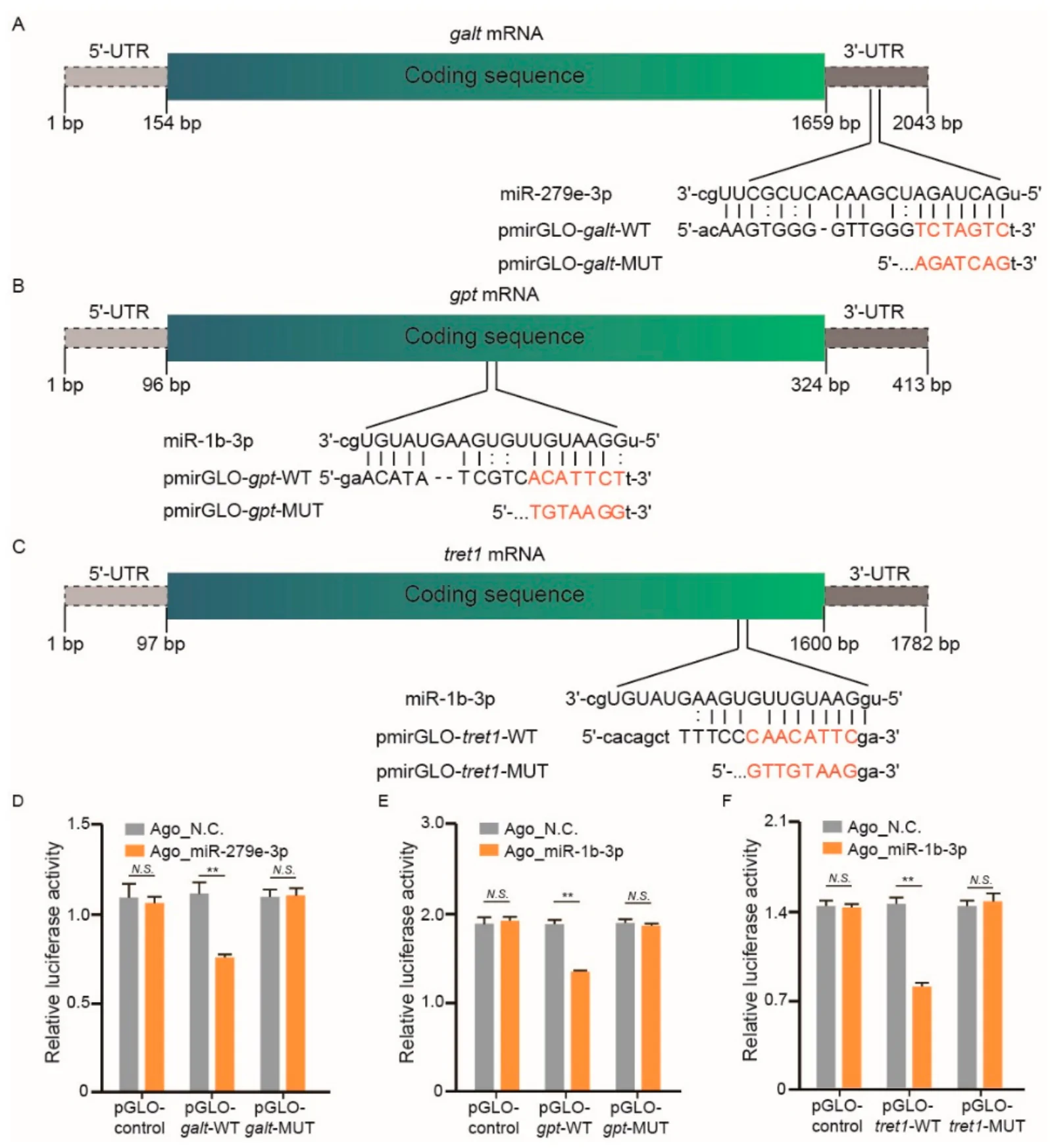
Validation of the binding between miR-279e-3p/miR-1b-3p and *galt*, *gpt*, and *tret1*. Prediction of miR-279e-3p and miR-1b-3p binding sites targeting *galt* (**A**), *gpt* (**B**), and *tret1* (**C**) and site-directed mutation of the pmirGLO plasmid. The red font indicates the base sequences before and after mutation. (**D**,**E**,**F**) Dual-luciferase reporter assay for miR-279e-3p and miR-1b-3p. Significance values were calculated for each comparison pair using *t*-test. ** represents *p* < 0.01, and *N.S.* denotes no significance.

Moreover, the binding regulatory sequences of miR-279e-3p and miR-1b-3p were predicted to be located within the 3′-UTR of *galt* and CDS sequences of *gpt* and *tret1* using the RNAhybrid tool, respectively (Figure 5A–C). To validate the roles of these binding sites in regulating expression of the target genes, the predicted seed sequences, including TCTAGTC of *galt* with miR-279e-3p, ACATTCT of *gpt* with miR-1b-3p, and CAACATTC of *tret1* with miR-1b-3p, were mutated to AGATCAG, TGTAAGG, and GTTGTAAG, respectively. Further analysis showed no significant difference in relative luciferase activity after transfection with the mutant and blank plasmids together with either the Ago_miRNAs or Ago_N.C. (Figure 5D–F). However, co-transfection of pmirGLO-*galt*-WT, pmirGLO-*gpt*-WT, and pmirGLO-*tret1*-WT with the corresponding Ago_miRNAs significantly (*p*-values < 0.05) resulted in the decreasing relative luciferase activity, which decreased by 34.74%, 26.32%, and 44.83%, compared to the Ago_N.C. groups, respectively. These results determined the targeted relationship of miR-279e-3p and miR-1b-3p with *galt*, *gpt*, and *tret1*.

## 4. Discussion

This study identified AARmiRNAs, potential target genes and KEGG pathways, uncovering a novel regulatory network associated with freshwater adaptation in *A. leii* larvae. The network was primarily related to energy metabolism, defense (detoxification) response, neural signal transduction, and growth and development in aquatic fireflies. In particular, energy metabolism was dominantly regulated by AARmiRNAs in this study, indicating that an important strategy by which aquatic fireflies adapt to fresh water, often characterized by hypoxic conditions, is the optimization of nutrients and energy metabolism [32,33]. Indeed, as a typical energy production process depending on aerobic metabolism, oxidative phosphorylation was a target of multiple AARmiRNAs. In addition, *cox5a*, co-targeted by miR-iab-8-5p and miR-iab-8 in this analysis, is involved in the oxidative phosphorylation pathway. Previous studies have demonstrated that COX5A can flexibly regulate cellular energy production depending on metabolic demand and oxygen availability [34,35]. The 2-oxocarboxylic acid metabolism pathway was regulated by the largest number of AARmiRNAs in this study. 2-Oxocarboxylic acids, also called alpha-keto acids, are important rate-limiting intermediates in the tricarboxylic acid cycle for energy production. These results suggested that AARmiRNAs likely controlled 2-oxocarboxylic acid levels in firefly cells by targeting 2-oxocarboxylic acid metabolism, regulating energy production. The valine, leucine and isoleucine biosynthesis and pantothenate and CoA biosynthesis pathways could be connected with the 2-oxocarboxylic acid metabolism pathway via *carm1* regulated by miR-31a. These three pathway connections constituted the biggest metabolic process regulated by AARmiRNAs. Furthermore, *carm1* is required for basal mitochondrial turnover and autophagic clearance, and it drives mitophagy during muscle atrophy [36], indicating that miR-31a governed *carm1*-mediated mitochondrial energy metabolism in freshwater adaptation of fireflies. The *syx1A* gene was targeted by miR-2944c-5p in the insulin secretion pathway, and it regulates the fusion of insulin granules during the early and sustained phases of insulin secretion [37]. Insects secrete insulin-like peptide, which is functionally similar to mammalian insulin [38], enhancing the storage of carbohydrates and lipids [39].

As a typical defense/detoxification response pathway, drug metabolism—cytochrome P450 is frequently reported in aquatic fireflies under water pollutants, such as benzo(a)pyrene [40,41]. Here, two AARmiRNAs (i.e., miR-184b and miR-72) regulated *impdh2* and *cyp3a4*, respectively, thereby influencing the drug metabolism—cytochrome P450 pathway; it has been reported that hypoxia adaptations are regulated by this pathway in aquatic fireflies via balancing oxygen use [16]. In particular, by doubling as a nucleotide biosynthetic enzyme or transcription factor, *impdh2* can either enable or restrict cell proliferation in *Drosophila*, and nuclear IMPDH accumulates following oxidative stress [42], with increasing DNA damage accumulation [43]. IMPDH2 also mediates PARP1 (poly ADP-ribose polymerase)-dependent DNA damage repair [43]. Therefore, miR-184b likely controls cell proliferation activity and the DNA damage response by regulating IMPDH levels in aquatic fireflies under oxidative stress in fresh water. CYP3A4, a key member of the CYP450 superfamily, participates in the oxidative stress response under hypoxia, contributing to the depletion of reactive oxygen species [44]. These results suggested that miR-72 controls the oxidative stress response resulting from aquatic hypoxia by regulating CYP450-mediated defense pathways in aquatic fireflies.

In this study, the AARmiRNAs, miR-31b-5p and miR-31a, regulated two pathways involved in neural signal transduction by targeting *gng10* and *gba*, respectively. As a member of the serotonergic synapse pathway, the GNG10 protein is a key component of olfactory signal transduction, as most olfactory receptors that encode G protein-coupled receptors require a heterotrimeric G protein composed of *α*, *β*, and *γ* subunits to initiate intracellular signal cascades, ultimately leading to olfactory perception in animals [45]. Variants in the *gba* gene, which encodes the lysosomal enzyme glucocerebrosidase, are the most common genetic abnormalities related to human Parkinson’s disease phenotypes, including motor, cognitive, olfactory and psychiatric symptoms, and *gba* mutations contribute to olfactory dysfunction [46,47]. These results suggest that AARmiRNAs regulate neural signal transduction pathways by targeting genes encoding G proteins and glucosylceramidases, likely impacting underwater living performance, such as olfactory sensitivity, motility, and tactile sensitivity.

This study identified a miRNA-based regulatory network associated with the development and lifespan of aquatic fireflies in freshwater adaptation. The hedgehog signaling pathway-fly is a signaling cascade first identified in *Drosophila*, with functions in developmental processes in metazoans [48]. The longevity regulating pathway is involved in prolongation of the lifespan of *Caenorhabditis elegans* [49]. In particular, the study results identified three gene members regulated by AARmiRNAs in the hedgehog signaling pathway-fly, including *galt*, *gpx*, and *slit3* targeted by miR-279e-3p, miR-31, and miR-71a-5p, respectively. Compared with those for a wild-type model, a longer time-to-pregnancy and growth restriction were detected in a *galt*-deficient model [50], showing that miR-279e-3p may be a key factor in the regulation of growth in aquatic fireflies. GPX is required for normal photoreceptor development and survival in humans and mice [51,52], suggesting that miR-31 regulates photoreceptor development in aquatic fireflies in response to decreased light in fresh water. The *slit3* gene mediates osteogenesis and angiogenesis in mammalian taxa [53,54,55]. Regulatory effects of miR-71a-5p on *slit3* found in this study likely contribute to the development of the circulatory system and insect exoskeleton to cope with hypoxia and water pressure. Collectively, these findings emphasize the importance of AARmiRNAs that regulate development-related pathways in aquatic fireflies. In addition, *akt1*, a member of the protein kinase family, was identified as a target of miR-iab-8 in the longevity regulating pathway. The knockout of *akt1* causes a progressive mass reduction in mice skeletal muscle, impairment of locomotive functions, and reduced lifespan [56,57]. The AARmiRNA, miR-iab-8, likely contributed to motor function and the normal lifespan of fireflies in aquatic adaptation.

## 5. Conclusions

This study documented several candidate AARmiRNAs that potentially regulate target genes and pathways associated with energy metabolism, defense/detoxification response, neural signal transduction, and development and lifespan in aquatic fireflies. Regulatory functions of these AARmiRNAs were linked to the responses of aquatic fireflies to ecological challenges in fresh water.

## Figures and Tables

**Table 1:** The miRNAs–target–KEGG pathway regulatory network.

MiRNAs	KEGG Pathways
miR-279e-3p	Oxidative phosphorylation
	Hedgehog signaling pathway-fly
miR-316	2-Oxocarboxylic acid metabolism
miR-71a-5p	Hedgehog signaling pathway-fly
miR-31	Oxidative phosphorylation
miR-31a	Pantothenate and CoA biosynthesis
	2-Oxocarboxylic acid metabolism
	Sphingolipid metabolism
miR-iab-8-5p	Oxidative phosphorylation
	2-Oxocarboxylic acid metabolism
miR-iab-8	Oxidative phosphorylation
	2-Oxocarboxylic acid metabolism
	Longevity regulating pathway
miR-31b-5p	Serotonergic synapse
miR-1b-3p	2-Oxocarboxylic acid metabolism
miR-184b	2-Oxocarboxylic acid metabolism
	Drug metabolism—cytochrome P450
miR-72	Drug metabolism—cytochrome P450
miR-2944c-5p	Insulin secretion

## Data Availability

All the raw data reported in this paper have been deposited in the Genome Sequence Archive in the National Genomics Data Center, China National Center for Bioinformation (accession number: CRA030551).

## Supplementary Materials

The following supporting information can be downloaded at https://www.mdpi.com/article/10.3390/insects17080845/s1. Table S1. Primers used in qRTPCR. Table S2. Primers used for vector construction. Table S3. Summary of small RNA-seq information for each sample. Table S4. List of aquatic adaptation-related miRNAs in aquatic *A. leii.* Table S5. List of enriched KEGG pathways for target genes of aquatic adaptation-related miRNAs of *A. leii*. Figure S1. PCA scatterplot scores for miRNA expression of four groups (ALA, ALL, PPA, PPL). Note: ALA: *A. leii* adult miRNAome, ALL: *A. leii* larvae miRNAome, PPA: *P. praetexta* adult miRNAome, PPL: *P. praetexta* larvae miRNAome.
