# Supplementary material for: Identification and Potential Functions of MicroRNAs Involved in Freshwater Adaptation in Aquatic Firefly Aquatica leii
Source: Insects. 2026 Aug 14;17(8):845. doi: 10.3390/insects17080845 (PMC13513562; doi:10.3390/insects17080845)
Supplement: Supplementary file 1 [file insects-17-00845-s001.zip › insects-4355005-supplementary.pdf]

Supplementary Materials

Supplementary methods

1. RNA extraction

The whole-body samples from larval and adult stages were separately homogenized in 2 mL of TRIzol (Invitrogen, Carlsbad, CA, USA) per 50–100 mg of tissue. The homogenized samples were incubated for 10 min at 25 ℃. Total RNA was purified from these samples using an established method for fireflies [40,41]. Residual genomic DNA was then digested using the RNase-free DNase Set (Qiagen, Hilden, Germany), following the manufacturer's instructions. RNA purity and integrity were assessed using a NanoDrop ND-1000 spectrophotometer (Thermo Scientific, Waltham, MA, USA). We adjusted the total RNA preparations from each developmental stage to a working concentration of 250 ng/μL with RNase-free water.

2. Preperation of sRNA-seq libraries

The libraries used for sRNA-seq were constructed using the Small RNA-Seq Library Prep Kit (Lexogen, Vienna, Austria). In brief, RNA segments of various sizes were separated by PAGE to filter small RNAs, and sequences in length range of 18–30 nt were selected. A connection system for adaptor ligation was prepared, mixed, and centrifuged, and the ligation reaction was continued at 25 ℃ for 1 h. A reverse transcription system was prepared for PCR amplification, generating double-stranded DNA. PAGE and extraction were used to purify PCR products, which were dissolved in EB solution. Finally, the prepared small RNA-sequencing libraries were input into the Agilent 2100 Bioanalyzer (Agilent, Santa Clara, CA, USA) and ABI StepOnePlus Real-Time PCR System (Applied Biosystems, Waltham, MA, USA) to assess the quality.

3. Expression analysis of miRNAs using qRTPCR

Total RNA was extracted from the samples (whole bodies) following methods described in 2.2 section. To validate the *in vivo* miRNA-mRNA relationship, we detected expression levels of miR-279e-3p-*galt* and miR-1b-3p using qRTPCR

analysis from samples in determination experiments of miRNA-mRNA relationship, as previously described [41], with minor modification. Specific qRTPCR primers were designed using Beacon Designer 7 (Table S1). RNase-free water was added to dilute the concentration of cDNA to 100 ng/μL. Fast SYBR™ Green Master Mix (Takara, Japan) was used for the qRTPCR, which was performed in an ABI 7300 Fast Real-Time PCR System (Applied Biosystems, USA). A negative control without cDNA template was included, and a melting curve was fit to test the quality of each reaction. The U6 snRNA gene was selected as an internal reference gene for miRNA quantification. For each reaction, three technical replicates and three biological replicates were performed.

4. Expression analysis of mRNA using qRTPCR

The purified RNA was similarly used in expression analysis of miRNAs. Specific qRTPCR primers of *galt*, *gpt*, and *tret1* were designed using Beacon Designer 7 (Table S1). Synthesis of the first-strand cDNA and qRTPCR analysis was performed according to methods used in our previous studies [40]. The elongation factor 1A (EF1A) gene was used as a housekeeping gene. A negative control without cDNA template was included, and a melting curve was fit to test the quality of each reaction.

5. Statistics in qRTPCR analysis

Data was visualized with the SDS tool (version 2.0) in the ABI 7300 Real-Time PCR system. Gene expression normalization and statistics were conducted based on the $2^{-\Delta\Delta Ct}$ method [57]. The significance of differences in comparison of pair was assessed using *t*-test in IBM SPSS statistics 22.

Supplementary tables and figures

Table S1. Primers used in qRTPCR.

MiRNAs/Unigene ID	Primer name	Primer sequence (5'-3')
miR-279e-3p	Stemloop primer	GTCGTATCCAGTGCAGGGTCCGAGGTATTCGCACTGGATACGACGCAAGC
	Forward primer	ACGCCAGTGACTAGATCGAACA
miR-1b-3p	Stemloop primer	GTCGTATCCAGTGCAGGGTCCGAGGTATTCGCACTGGATACGACGCACAT
	Forward primer	AGCGCACTGGAATGTTGTGAAG
U6	Stemloop primer	GTCGTATCCAGTGCAGGGTCCGAGGTATTCGCACTGGATACGACAAAAAT
	Forward primer	AACAAGGTTCTTGCTTCGGC
Universal reverse primer		GTCGTATCCAGTGCAGGGT
c132225.graph_c1 (*galt*)	Forward primer	GGAGTGGGTCAGAGAAACCG
	Reverse primer	CAACCCATCATAGCGCCTCT
c46235.graph_c0 (*gpt*)	Forward primer	ACTGGTCTGTTTTTGTGCGC
	Reverse primer	TCTCCATGTAACCTCCCCGT
c126480.graph_c0 (*tret1*)	Forward primer	TGGTGCGCTTGGAAGTTACT
	Reverse primer	ATACACGGGCGTTTCTGGTT
c78803.graph_c0 (*ef1a*)	Forward primer	TCATCTGCTTGACACCGAGG
	Reverse primer	ACGTCGCCATCCTCATCATC

Table S2. Primers used for vector construction.

Gene name	Primer name	Sequence (5'-3')
galt	SacI-*galt*-F	CGAGCTCTGAAAACAACGTTCAGAAGAAG
	XhoI-*galt*-R	CCGCTCGAGGCCTATACCTAAACTTT
gpt	SacI-*gpt*-F	CGAGCTCATGGAATATGCGGTCCGTGG
	XhoI-*gpt*-R	CCGCTCGAGGCCCTTTGTTTCACGTCGTC
tret1	SacI-*tret1*-F	CGAGCTCGGCTTCCAATTGCGTGTCTC
	XhoI-*tret1*-R	CCGCTCGAGACGCAAAGACACAACAACCT
pmirGLO	pmirGLO-F	ACACGGTAAAACCATGAC
pmirGLO	pmirGLO-F	TCCAAACTCATCAATGTATC

Table S3. Summary of small RNA-seq information for each sample.

Category	*Pyrocoelia praetexta*						*Aquatica leii*					
	**PPA1**	**PPA2**	**PPA3**	**PPL1**	**PPL2**	**PPL3**	**ALA1**	**ALA2**	**ALA3**	**ALL1**	**ALL2**	**ALL3**
Total raw tags	12,085,330	11,674,569	10,498,921	12,234,678	12,496,264	11,729,602	12,159,955	12,294,291	9,892,356	10,648,451	11,774,529	11,923,318
Total clean tags	9,928,974	9,953,761	8,482,841	8,523,545	11,901,855	10,258,880	10,437,250	5,576,804	7,560,287	8,539,858	8,517,432	5,677,709
Clean reads ratio (%)	82.16	85.26	80.80	69.67	95.24	87.46	85.83	45.36	76.43	80.20	72.34	47.62
Known miRNAs	183	201	191	184	183	180	186	191	203	186	182	180

Table S4. List of aquatic adaptation-related miRNAs in aquatic *A. leii*.

MiRNA id	Mature sequence	Log_2(fold change (ALA/ALL))	Up/Down-Regulation	*FDR*
miR-279e-3p	UGACUAGAUCGAACACUCGCUUGC	9.91	Up	3.17E-19
miR-2944b-5p	GAAGGAACUCCCGGUGUGAUAUG	7.41	Up	9.79E-09
miR-1b-3p	UGGAAUGUUGUGAAGUAUGUGC	6.71	Up	3.89E-07
miR-71a-5p	UGAAAGACGAUGGUAGUGAGA	6.34	Up	2.84E-06
miR-iab-8-5p	UUACGUAUACUGAAGGUAUACCGGAC	6.00	Up	3.81E-05
miR-31a	GGCAAGAUGUCGGCAUAGCUGA	4.69	Up	3.56E-03
miR-276a	UAGGAACUUCAUACCGUGCUCU	4.20	Up	1.06E-02
miR-iab-8	UUACGUAUACUGAAGGUAUACCG	3.99	Up	2.17E-02
miR-31	AGGCAAGAUGUUGGCAUAGCU	2.76	Up	1.84E-204
miR-31b-5p	UGGCAAGAUGUCGGAAUAGCUG	2.23	Up	7.63E-03
miR-184b	UGGACGGAGAACUGAUAGGGC	2.16	Up	6.56E-05
miR-316	UGUCUUUUUCCGCUUUGCUGC	1.67	Up	5.85E-03
miR-2779	AUCCGGCUCGAAGGACCA	1.62	Up	1.02E-02
miR-927a-5p	UUUAGAAUUCCUACGCUUUA	1.26	Up	2.17E-02
miR-71	UGAAAGACAUGGGUAGUGA	1.23	Up	1.08E-06
miR-998	UAGCACCAUGGGAUUCAGCUCA	-1.22	Down	3.31E-06
miR-87a	GUGAGCAAAGUUUCAGGUGUGC	-3.10	Down	6.65E-03
miR-72	AGGCAAGAUGUUGGCAUAGCUGA	-3.82	Down	3.30E-02
miR-1985	UGCCAUUUUUAUCAGUCACUGUG	-4.80	Down	2.92E-03
miR-2944c-5p	GAAGGAACUUCUGGUGUGAUAUG	-6.63	Down	3.07E-10

Table S5. List of enriched KEGG pathways for target genes of aquatic adaptation-related miRNAs of *A. leii*.

**Pathway**	**Pathway id**	***FDR***
2-Oxocarboxylic acid metabolism	ko01210	1.40E-03
Hedgehog signaling pathway - fly	ko04341	3.73E-03
Insulin secretion	ko04911	4.70E-03
Serotonergic synapse	ko04726	4.70E-03
Oxidative phosphorylation	ko00190	4.70E-03
Pantothenate and CoA biosynthesis	ko00770	7.18E-03
Valine, leucine and isoleucine biosynthesis	ko00290	1.72E-02
Longevity regulating pathway	ko04211	2.67E-02
Drug metabolism - cytochrome P450	ko00982	2.80E-02
Sphingolipid metabolism	ko00600	4.61E-02

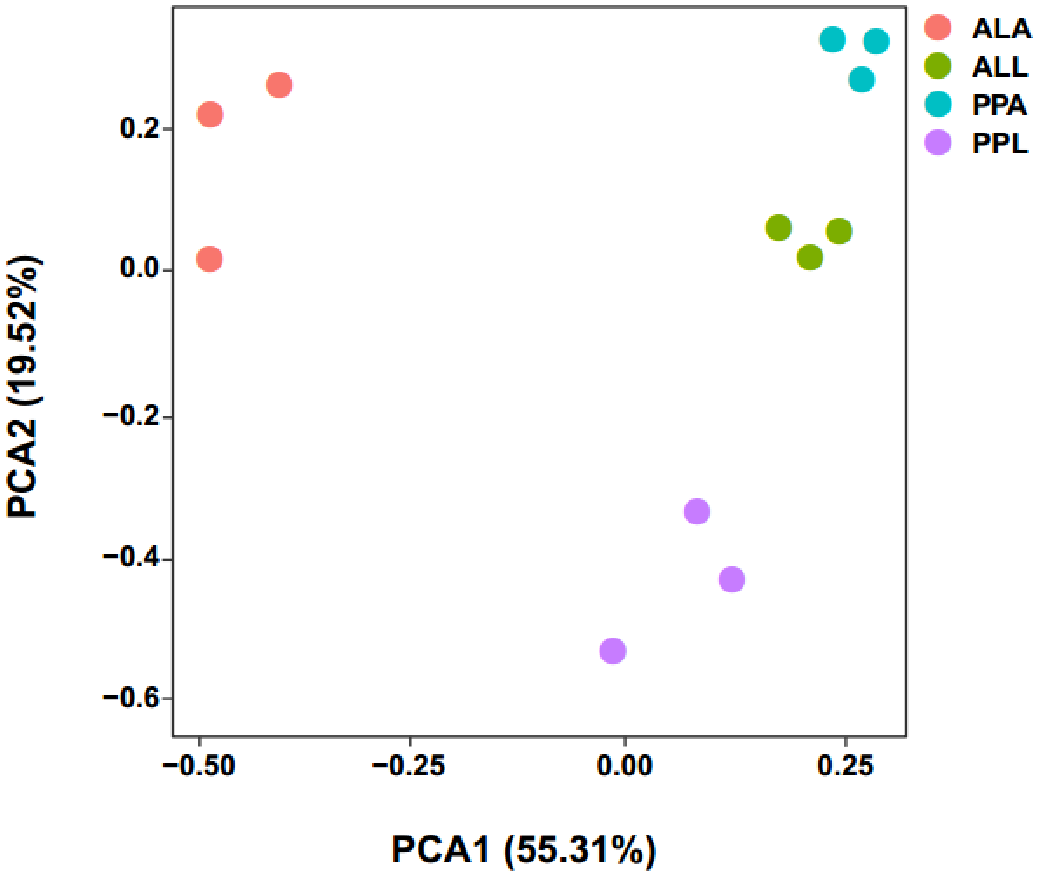

Figure S1. PCA scatterplot scores for miRNA expression of four groups (ALA, ALL, PPA, PPL). Note: ALA: *A. leii* adult miRNAome, ALL: *A. leii* larvae miRNAome, PPA: *P. praetexta* adult miRNAome, PPL: *L. praetexta* larvae miRNAome.
